# CHA_2_DS_2_-VASc score is useful in predicting poor 12-month outcomes following myocardial infarction in diabetic patients without atrial fibrillation

**DOI:** 10.1007/s00592-016-0877-6

**Published:** 2016-06-23

**Authors:** Bartosz Hudzik, Janusz Szkodziński, Michal Hawranek, Andrzej Lekston, Lech Poloński, Mariusz Gąsior

**Affiliations:** Third Department of Cardiology, Silesian Center for Heart Disease, SMDZ in Zabrze, Medical University of Silesia in Katowice, Curie-Sklodowska 9, 41-800 Zabrze, Poland

**Keywords:** STEMI, Diabetes mellitus, Risk score, CHA_2_DS_2_-VASc, Prognosis

## Abstract

**Aims:**

TIMI risk score and GRACE risk model are widely available and accepted scores for risk assessment in STEMI patients and include predictors of poor outcomes. CHA_2_DS_2_-VASc is a validated score for predicting embolic/stroke risk in patients with non-valvular atrial fibrillation. Its components contribute to the worse prognosis following myocardial infarction. The advantage of the CHA_2_DS_2_-VASc score in comparison with other risk scores is that it provides a comprehensive, fast, and simple method for physicians in risk evaluation that requires no calculators or computers. Therefore, we have set out to examine the prognostic significance of CHA_2_DS_2_-VASc score following STEMI in diabetic patients without AF.

**Methods:**

A total of 472 patients with diabetes mellitus and STEMI undergoing primary PCI were enrolled. Based on the estimated CHA_2_DS_2_-VASc score, the study population was divided into three groups: group 1 (*N* = 111) with a moderate CHA_2_DS_2_-VASc score of 2 or 3; group 2 (*N* = 257) with a high CHA_2_DS_2_-VASc score of 4 or 5; and group 3 (*N* = 104) with a very high CHA_2_DS_2_-VASc score of 6 or higher.

**Results:**

In diabetic patients with STEMI, the median of CHA_2_DS_2_-VASc score was 4 (interquartile range 3–5). In-hospital mortality rate was similar across three groups. CHA_2_DS_2_-VASc score was not a risk factor of in-hospital mortality. ROC analysis revealed good diagnostic value of CHA_2_DS_2_-VASc score in predicting long-term mortality (AUC 0.62 95 % CI 0.57–0.66 *P* = 0.0003) and stroke (AUC 0.75 95 % CI 0.71–0.79 *P* = 0.0003), but no value in predicting long-term myocardial infarction. CHA_2_DS_2_-VASc score was an independent predictor of 12-month mortality and stroke. One-point increment in CHA_2_DS_2_-VASc score was associated with an increase in the risk of 12-month death by 24 % and for 12-month stroke by 101 %.

**Conclusions:**

In diabetic patients with STEMI and no previous AF, median CHA_2_DS_2_-VASc score was high (4 points) and predicted 12-month death and stroke. However, it failed to predict in-hospital death and 12-month MI. CHA_2_DS_2_-VASc score had a similar discrimination performance in predicting 12-month mortality as TIMI risk score and a better discrimination performance in predicting 12-month stroke than TIMI risk score. Thus, it can serve as an additive tool in identifying high-risk patients that require aggressive management.

## Introduction

Outcomes following ST-segment elevation myocardial infarction (STEMI) have improved over the past two decades. Nevertheless, survivors of STEMI have an elevated risk of subsequent vascular events, including stroke and 12-month mortality approaches 10 % [1]. Risk stratification following STEMI plays an essential role in managing patients and is recommended prior to discharge by the current guidelines. TIMI risk score and GRACE risk model are widely available and accepted scores for risk assessment and include predictors of poor outcomes which were established in large databases of MI patients [2–4]. The algorithms aid clinicians in assessing prognosis and may therefore be useful in guiding management. These two models are both fitted for predicting short- and long-term prognosis and assess the risk in a two-step process which includes: (1) stratifying patients at admission based on demographics, physical examination, presenting signs, and initial laboratory and angiographic data, and (2) identifying long-term risk based on the development of post-event complications. These scores were designed to be simple and practical, although they comprise numerous variables.

Evidence suggests that diabetes mellitus (DM) is among the most important risk factors of poor outcome following STEMI. DM is one of the components of the CHA_2_DS_2_-VASc score. Such a high incidence of thromboembolic events observed in these clinical subsets may be attributable to the DM-related prothrombotic state [5]. Generally, the diagnosis of DM is pivotal to assess cardiovascular risk and to guide therapy and lifestyle modification. Particularly, this topic is especially relevant for those patients who have already experienced a major cardiovascular event [6]. In addition, the presence of coronary artery disease is a prognostic factor in DM complications [7, 8]. Accurate risk assessment together with regular follow-up may result in a lower glycemic burden and a lower rate of vascular complications [9].

CHADS_2_ and the more recent CHA_2_DS_2_-VASc are two validated scores for predicting embolic/stroke risk in patients with non-valvular atrial fibrillation (AF) [10, 11]. They aid us in guiding antithrombotic therapy in AF patients. The individual score components not only predict AF-associated stroke risk but also are linked to the development of AF [12–14]. The CHADS_2_ and CHA_2_DS_2_-VASc scores have been reported to identify post-STEMI patients at high risk of AF and ischemic stroke [14].

In addition to predicting the risk of stroke/embolic events in AF patients, CHA_2_DS_2_-VASc score components (i.e., increasing age, hypertension, diabetes mellitus, prior cardiovascular events) are traditional risk factors that are associated with atherosclerosis, coronary artery disease, and contribute to the worse prognosis following myocardial infarction (MI). However, only a few studies have examined the prognostic value of CHA_2_DS_2_-VASc following MI [15, 16], and no studies have been found to evaluate CHA_2_DS_2_-VASc among STEMI or diabetic populations exclusively.

The advantage of the CHA_2_DS_2_-VASc score in comparison with other risk scores is that it provides a comprehensive, fast, and simple method for physicians in risk evaluation that requires no calculators or computers. Therefore, we have set out to examine the prognostic significance of CHA_2_DS_2_-VASc score following STEMI in diabetic patients. In the interest of averting a possible effect of AF on the outcomes, we have performed the analysis in a population of patients without AF.

## Materials and methods

The study conforms to the Declaration of Helsinki. Informed consent for data analysis was obtained from the patients according to the Polish law on patients’ rights regarding data registration. Approval for analyzing recorded data was waived by the local bioethics committee on human research given the retrospective nature of the study. Patients admitted with diagnosis of STEMI, within 12 h from symptom onset were enrolled in the study. Patients with a history of or newly diagnosed AF were excluded. This is a single-center, cross-sectional, retrospective study.

A total of 472 patients with diabetes mellitus and STEMI undergoing primary PCI were enrolled. Based on the estimated CHA_2_DS_2_-VASc score, the study population was divided into three groups: group 1 (*N* = 111) with a moderate CHA_2_DS_2_-VASc score of 2 or 3; group 2 (*N* = 257) with a high CHA_2_DS_2_-VASc score of 4 or 5; and group 3 (*N* = 104) with a very high CHA_2_DS_2_-VASc score of 6 or higher.

The components of CHA_2_DS_2_-VASc score include: congestive heart failure (1 point), hypertension (1 point), age >75 years (2 points), diabetes mellitus (1 point), history of stroke (2 points), history of vascular disease (1 point), age >65 years (1 point), and female sex (1 point) [11]. The history of myocardial infarction was regarded as ‘vascular disease’, and the current STEMI was counted as 1 point. As the study investigated only diabetic patients, all patients have received a minimum score of 2 points.

All patients received loading doses of antiplatelet medications (aspirin, clopidogrel) before admission to our hospital (either in the referring hospital or ambulance) according to the guidelines. Diabetes mellitus was defined as: (a) preexisting condition diagnosed before STEMI (patients on insulin, oral glucose-lowering drugs, or on a diet) and (b) newly diagnosed diabetes mellitus based on fasting plasma glucose (FPG) ≥7.0 mmol/L or 2-h plasma glucose ≥11.1 mmol/L during an oral glucose tolerance test (OGTT) [17]. To avoid acute hyperglycemia, FPG was taken into consideration after the third day of hospital stay. For that reason, OGTT was performed on day four of hospital stay or later. STEMI was defined as: (1) ST-segment elevation consistent with MI of at least 2 mm in contiguous precordial leads and/or ST-segment elevation of at least 1 mm in two or more limb leads or new left bundle branch block, and (2) positive cardiac necrosis markers: CK-MB mass (upper limit of normal: 4.9 mg/mL) and/or troponin T (upper limit of normal: 0.014 ng/mL). Patients received 300 mg of acetylsalicylic acid (ASA) loading dose and 600 mg of clopidogrel loading dose, followed by 75 mg of ASA maintenance dose and 75 mg of clopidogrel maintenance dose [18]. Coronary angiography and percutaneous coronary interventions were performed using standard protocols and guidelines. A culprit lesion was described in the presence of an acute occlusion, intraluminal filling defects (or thrombus), ulcerated plaques, dissection, or intraluminal flaps. Successful PCI was defined as a post-procedural residual-diameter stenosis <30 %, with TIMI 3 flow in the infarct-related artery and no procedural complications.

All patients were scheduled for an elective 12-month clinical follow-up. We clinically monitored the patients for cardiovascular events. The major adverse cardiac and cerebrovascular events (MACCEs) included death, rehospitalization for myocardial infarction, and stroke.

### Statistical analysis

Quantitative data are presented as means ± standard deviations (SDs) or medians with interquartile ranges (lower and upper quartiles). Qualitative data are presented as frequencies. The Shapiro–Wilk test was used to determine whether random samples came from a normal distribution. The Chi-square test with Yates’ correction was used to compare categorical variables. One-way analysis of variance (ANOVA) and Kruskal–Wallis ANOVA tests were used to compare continuous variables between groups for variables normally and not normally distributed, respectively. In-hospital and one-year survival was estimated with the Kaplan–Meier method and compared with the log-rank test. The relationship between CHA_2_DS_2_-VASc score and clinical variables was evaluated by Spearman’s rank correlation coefficient. Receiver-operating characteristic (ROC) curves were estimated for CHA_2_DS_2_-VASc score. ROC analysis was used to determine the cutoff values of CHA_2_DS_2_-VASc score to predict in-hospital and 12-month mortality, myocardial infarction, and stroke. The effects of the clinical and angiographic variables on the in-hospital and 12-month mortality were assessed using the multivariate Cox proportional hazard regression models with the results expressed as hazard ratios (HRs) and 95 % confidence intervals (CIs). Variables with a significant influence on mortality in univariate analysis were entered into the multivariate model. These included: history of myocardial infarction, left ventricular ejection fraction (per 1 % increment), success of PCI (final TIMI 3 flow) in the culprit vessel, cardiogenic shock, and time form symptom onset (per 1-h increment). Variables with a significant influence on 12-month stroke in univariate analysis were entered into the multivariate model. These included: history of myocardial infarction, anterior myocardial infarction on admission, and left ventricular ejection fraction (per 1 % increment).

A value of *P* < 0.05 was considered significant.

## Results

**Fig. 1:**
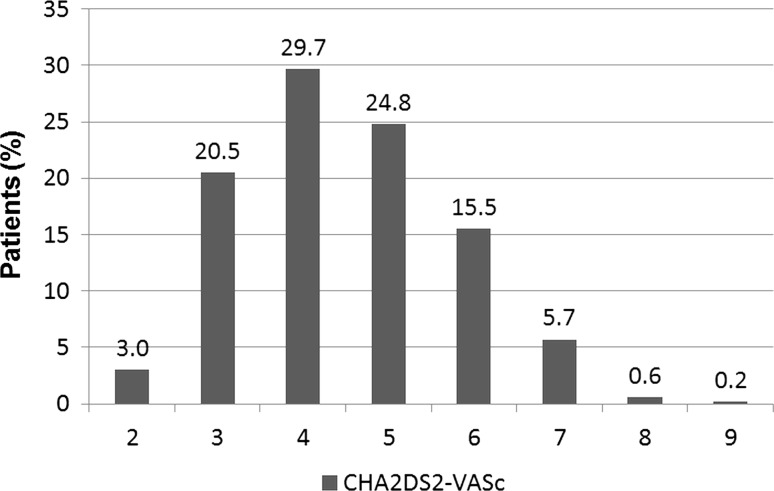
Number of patients falling in each CHA_2_DS_2_-VASc score category

**Fig. 2:**
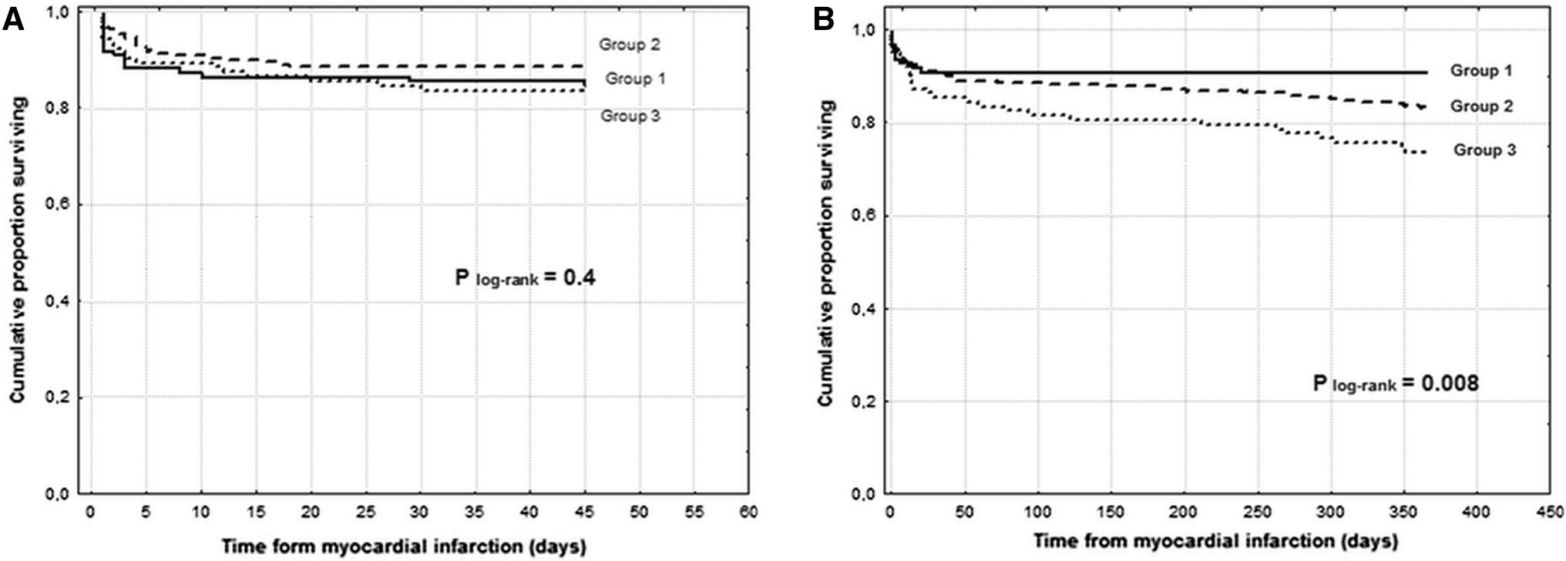
**a** Kaplan–Meier in-hospital survival curves. **b** Kaplan–Meier 12-month survival curves. Group 1 versus 2 *P* = 0.06; group 1 versus 3 *P* = 0.001; group 2 versus 3 *P* = 0.04

**Fig. 3:**
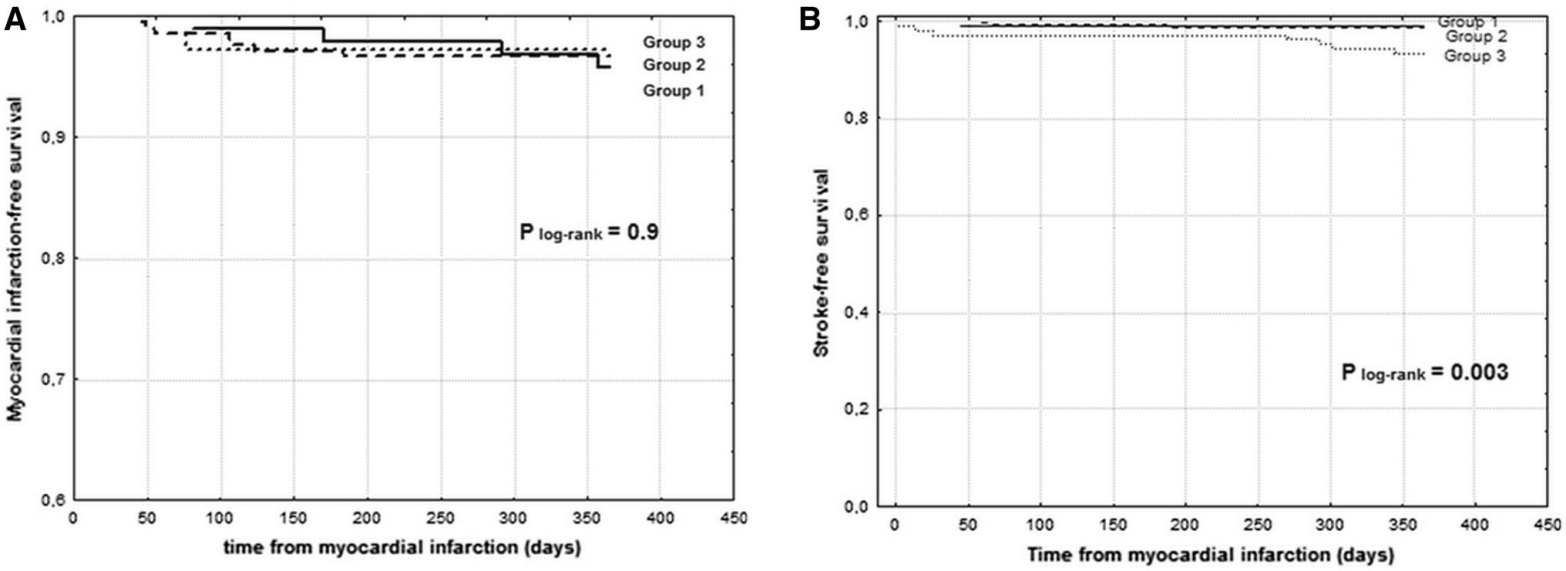
**a** Kaplan–Meier 12-month myocardial infarction-free survival curves. **b** Kaplan–Meier 12-month stroke-free survival curves. Group 1 versus 2 *P* = 0.8; group 1 versus 3 *P* = 0.02; group 2 versus 3 *P* = 0.03

**Fig. 4:**
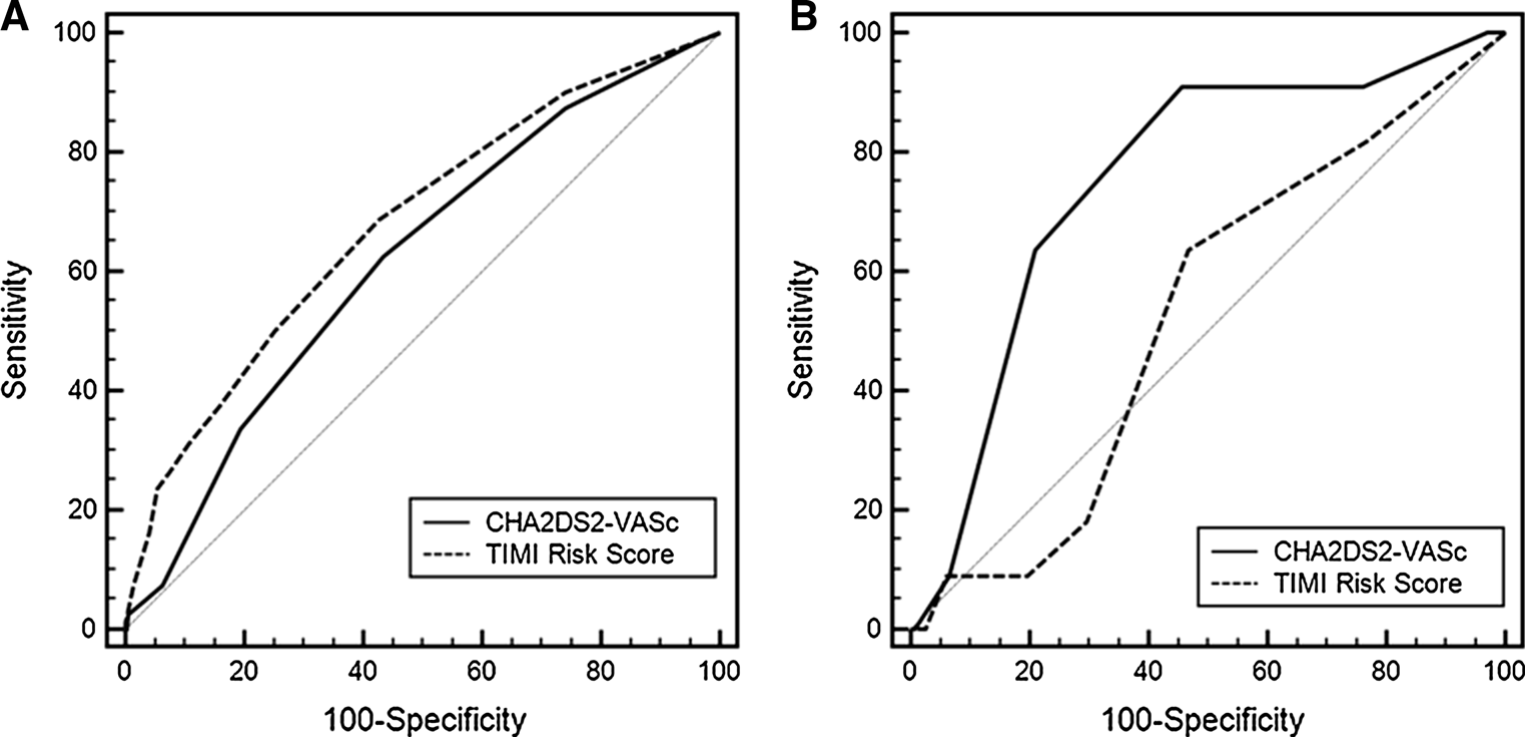
Receiver-operating characteristic (ROC) curves for the discrimination performance of CHA_2_DS_2_-VASc score and TIMI risk score for prediction of 12-month mortality (**a**) and 12-month stroke (**b**)

In diabetic patients with STEMI, the median of CHA_2_DS_2_-VASc score was 4 (interquartile range 3–5). The number of patients falling in each CHA_2_DS_2_-VASc score category is depicted in Fig. 1. Baseline clinical characteristics are featured in Table 1. Patients with moderate CHA_2_DS_2_-VASc score (group 1) were younger and more frequently men. Hypertension and a lower ejection fraction were more prevalent among patients with a high and very high CHA_2_DS_2_-VASc score (groups 2 and 3). Angiographic data are presented in Table 2. There was a trend toward a worse initial TIMI flow in group 3. Initial TIMI 3 flow was the least prevalent in group 3. Notwithstanding, final TIMI flow was similar in all three groups. ROC analysis demonstrated no value of CHA_2_DS_2_-VASc score in predicting in-hospital mortality AUC 0,503 (95 % CI 0.463–0.555) *P* = 0.83. In-hospital mortality rate was similar across three groups (Fig. 2a). CHA_2_DS_2_-VASc score was not a risk factor of in-hospital mortality (Table 3). During 12-month follow-up, there were 10 deaths (9.0 %) in group 1, 43 deaths (16.7 %) in group 2, and 27 deaths (16.2 %) in group 3 (P = 0.003) (Table 3; Fig. 2b). The rate of non-fatal myocardial infarction was similar across all groups, and the rate of stroke was higher in group 3 (Table 4; Fig. 3a, b). ROC analysis revealed good diagnostic value of CHA_2_DS_2_-VASc score in predicting 12-month mortality and stroke, but no value in predicting 12-month myocardial infarction (Tables 5, 6; Fig. 4a, b). CHA_2_DS_2_-VASc score was an independent predictor of 12-month mortality and stroke. One-point increment in CHA_2_DS_2_-VASc score was associated with an increase in the risk of 12-month death by 24 % and for 12-month stroke by 101 %. CHA_2_DS_2_-VASc score was positively correlated with age (Spearman R = 0.67 *P* < 0.0001) and TIMI risk score (Spearman *R* = 0.40 *P* < 0.0001), and negatively correlated with ejection fraction (Spearman *R* = 0.28 *P* < 0.0001), time to stroke during follow-up (Spearman *R* = −0.13 *P* = 0.003), and time to death during follow-up (Spearman *R* = −0.15 *P* = 0.001).

**Table 1:** Patients’ baseline and clinical characteristics

	Group 1 *N* = 111	Group 2 *N* = 257	Group 3 *N* = 104	*P*
Age, years (mean ± SD)	57 ± 8^†^	64 ± 8^†^	73 ± 6^†^	0.0001
Sex, men, *N* (%)	106 (95.5 %)	150 (58.4 %)	15 (14.4 %)	0.0001
Systemic hypertension, *N* (%)	41 (36.9 %)	198 (77.0 %)	100 (96.1 %)	0.0001
Anterior myocardial infarction, *N* (%)	34 (30.6 %)	89 (34.6 %)	36 (34.6 %)	0.7
Prior myocardial infarction, *N* (%)	34 (30.6 %)	78 (30.5 %)	32 (30.8 %)	0.9
Time from symptom onset, hours [median (interquartile range)]	5.0 (3.0–7.0)	4.5 (3.0–7.0)	4.5 (3.0–7.5)	0.3
Cardiogenic shock, *N* (%)	15 (13.5 %)	33 (12.8 %)	16 (15.4 %)	0.8
LVEF, (%) [median (interquartile range)]	50 (41–55)*	40 (35–46)*	40 (35–45)*	0.0001
Hospital stay, days [median (interquartile range)]	8 (6–11)	9 (6–12)	8 (6–12)	0.09
TIMI risk score	3 (2–4)^§^	3 (2–5)^§^	4 (3–6)^§^	<0.0001
HbA1c (%) [median (interquartile range)]	7.6 (7.0–8.0)	7.6 (6.9–8.0)	7.5 (6.9–8.2)	0.9
Admission glycemia (mmol/l) [median (interquartile range)]	8.1 (7.0–11.4)	7.8 (6.8–10.2)	8.0 (6.2–10.3)	0.2
Fasting plasma glucose (mmol/L) [median (interquartile range)]	6.7 (5.3–7.8)	6.8 (5.8–7.9)	6.6 (5.2–8.3)	0.6
In-hospital death, *N* (%)	17 (15.3 %)	30 (11.7 %)	17 (16.3 %)	0.4

*SD* standard deviation, *LVEF* left ventricular ejection fraction

* Group 1 versus group 2 *P* < 0.001, group 1 versus group 3 *P* < 0.001, and group 2 versus group 3 *P* = 0.7

^†^Group 1 versus group 2 *P* < 0.0001, group 1 versus group 3 *P* < 0.0001, and group 2 versus group 3 *P* < 0.0001

^§^Group 1 versus group 2 *P* = 0.01, group 1 versus group 3 *P* < 0.0001, and group 2 versus group 3 *P* < 0.0001

**Table 2:** Angiographic findings

	Group 1 *N* = 111	Group 2 *N* = 257	Group 3 *N* = 104	*P*
Multivessel CAD, *N* (%)	49 (44.1 %)	117 (45.5 %)	47 (45.2 %)	0.6
Initial TIMI flow, *N* (%)				
0	63 (56.8 %)	150 (58.6 %)	56 (53.8 %)	0.08
1	18 (16.2 %)	46 (17.9 %)	18 (17.3 %)	
2	14 (12.6 %)	35 (13.7 %)	25 (24.0 %)	
3	16 (14.4 %)	26 (10.1 %)	5 (4.8 %)	
Initial TIMI 3 flow, *N* (%)	16 (14.4 %)	26 (10.1 %)	5 (4.8 %)	0.05
Final TIMI flow, *N* (%)				
0	5 (4.5 %)	17 (6.6 %)	8 (7.1 %)	0.8
1	1 (0.9 %)	3 (1.2 %)	2 (1.9 %)	
2	11 (10.0 %)	19 (7.4 %)	9 (8.7 %)	
3	94 (84.6 %)	218 (85.1 %)	85 (81.3 %)	
Final TIMI 3 flow, *N* (%)	94 (84.6 %)	218 (85.1 %)	85 (81.3 %)	0.7

**Table 3:** Predictors of in-hospital and 12-month mortality

In-hospital mortality						
	Unadjusted			Adjusted		
	HR	95 % CI	*P*	HR	95 % CI	*P*
CHA_2_DS_2_-VASc score (per one-point increment)	0.99	0.81–1.21	0.9	–	–	–

Twelve-month mortality						
	Unadjusted			Adjusted*		
	HR	95 % CI	*P*	HR	95 % CI	*P*
CHA_2_DS_2_-VASc score (per one-point increment)	1.31	1.11–1.54	0.001	1.24	1.06–1.45	0.008

Twelve-month myocardial infarction						
	Unadjusted			Adjusted		
	HR	95 % CI	*P*	HR	95 % CI	*P*
CHA_2_DS_2_-VASc score (per one-point increment)	0.78	0.52–1.16	0.22	–	–	–

Twelve-month stroke						
	Unadjusted			Adjusted**		
	HR	95 % CI	*P*	HR	95 % CI	*P*
CHA_2_DS_2_-VASc score (per one-point increment)	1.81	1.18–2.79	0.006	2.01	1.25–3.23	0.004

*HR* hazard ratio, *CI* confidence interval

* Adjusted for: history of myocardial infarction, left ventricular ejection fraction (per 1 % increment), successful percutaneous coronary intervention (final TIMI 3 flow) in the culprit vessel, cardiogenic shock, time form symptom onset (per 1-h increment), platelet distribution width (per 1fL increment), and platelet count (per 10^4^/mm^3^ increment)

** Adjusted for: history of myocardial infarction, left ventricular ejection fraction (per 1 % increment), anterior myocardial infarction during index hospitalization

**Table 4:** 12-month follow-up

	Group 1 *N* = 111	Group 2 *N* = 257	Group 3 *N* = 104	*P*
All-cause mortality, *N* (%)	10 (9.0 %)	43 (16.7 %)	27 (16.2 %)	0.003
Non-fatal myocardial infarction, *N* (%)	5 (4.5 %)	9 (3.5 %)	3 (2.9 %)	0.7
Stroke, *N* (%)	0 (0 %)	3 (1.1 %)	7 (6.7 %)	0.004

**Table 5:** Receiver-operating characteristics curves identifying the discrimination thresholds of CHA_2_DS_2_-VASc score for predicting 12-month mortality, non-fatal myocardial infarction, and stroke

	Cut off	AUC	95 % CI	Sensitivity (%)	Specificity (%)	PPV (%)	NPV (%)	*P*
All-cause mortality	>4	0.62	0.57–0.66	62	56	23	88	0.0003
Myocardial infarction		0.58	0.45–0.62					0.3
Stroke	>4	0.75	0.71–0.79	91	54	5	99	0.0003

**Table 6:** Comparison of the receiver-operating characteristics curves identifying the discrimination thresholds of CHA_2_DS_2_-VASc score and TIMI risk score for long-term hospital mortality and stroke

	Cut off	AUC	95 % CI
Twelve-month mortality			
CHA_2_DS_2_-VASc score	>4	0.67	0.63–0.71
TIMI risk score	>4	0.60	0.56–0.65
Difference between areas CHA_2_DS_2_-VASc score and TIMI risk score		0.06	
95 % CI		−0.03–0.15	
*P*		0.18	
Twelve-month stroke			
CHA_2_DS_2_-VASc score	>4	0.75	0.71–0.79
TIMI risk score	>4	0.52	0.48–0.57
Difference between areas CHA_2_DS_2_-VASc score and TIMI risk score		0.22	
95 % CI		0.06–0.38	
*P*		0.006	

## Discussion

In the current study, we have set out to investigate the utility of CHA_2_DS_2_-VASc score in predicting poor outcomes in patients with diabetes mellitus and STEMI. In particular, we have sought to determine whether CHA_2_DS_2_-VASc score commonly used for prediction of thromboembolic events in AF patients can be used as a risk assessment tool in STEMI patients without AF.

There are several major findings of the study. First and foremost, CHA_2_DS_2_-VASc score was high across the entire study population with a median of 4 points. Second, it had no influence on the in-hospital mortality. The third major finding was that CHA_2_DS_2_-VASc score, both unadjusted and adjusted for potential cofounders, correlated with 12-month stroke and all-cause mortality, but it had no influence on 12-month myocardial infarction. CHA_2_DS_2_-VASc score showed similar predictive value with respect to 12-month mortality as TIMI risk score. More importantly, it demonstrated a better predictive value with respect to 12-month stroke than TIMI risk score. And finally, CHA_2_DS_2_-VASc score showed a negative correlation with the time to subsequent stroke or death during follow-up.

CHADS_2_ score was developed to identify AF patients at risk for stroke or thromboembolic event and thus to guide anticoagulation therapy [10]. However, patients with an intermediate risk (CHADS_2_ score 1) presented a challenge in everyday practice because some of them were at low–intermediate risk and others were at high–intermediate risk. Consequently, CHA_2_DS_2_-VASc score was introduced to improve predictive value for thromboembolic events in patients at low and intermediate risk [11]. The utility of the score goes beyond the benefits of risk stratification for thromboembolic events. Apiyasawat et al. [19] reported it to be an independent prognostic marker of mortality following hospitalization for AF. All elements of the CHA_2_DS_2_-VASc score are essential risk factors in cardiovascular disease. Hypertension, diabetes mellitus, and congestive heart failure were all found to be predictors of poor outcomes following acute MI [20].

As pointed out in the introduction to this paper, there is little published reports on the utility of CHA_2_DS_2_-VASc score in risk stratification following acute MI. Poci et al. [21] analyzed 2335 patients with acute coronary syndromes and reported that CHADS_2_ score was associated with long-term mortality (HR 1.38 95 % CI 1.28–1.48 *P* < 0.0001) and stroke (HR 1.46 95 % CI 1.27–1.68 *P* < 0.0001). Kim et al. [15] performed a retrospective analysis of 15,681 patients with acute MI who were enrolled in the Korean Working Group in Acute Myocardial Infarction (KORMI) registry. They reported that CHA_2_DS_2_-VASc score was associated with long-term cardiac events (MI, all-cause death) (HR 1.384 *P* < 0.0001). Interestingly, they found that CHA_2_DS_2_-VASc score was a more important predictor in STEMI patients (HR 1.455 P < 0.0001) than in NSTEMI patients (HR 1.298 P = 0.048). Piccini et al. [22] proposed a modified R-CHA_2_DS_2_-VASc score by adding renal function parameters (glomerular filtration rate and urea), performance of a revascularization procedure, and history of atrial fibrillation. Based on that modified R-CHA_2_DS_2_-VASc score, a study showed a good calibration and high discriminative performance of that score in the prediction of post-discharge ischemic stroke and all-cause mortality [23]. Recently, Podolecki et al. [16] studied 2980 patients with acute coronary syndromes and no AF and reported that an increment of one point in the CHA_2_DS_2_-VASc score was independently associated with a 41 % increase in stroke risk and a 23 % increase in mortality rate (*P* < 0.001 for both). Our results agree with the aforementioned studies. We found that one-point increment in CHA_2_DS_2_-VASc score was associated with an increase in the risk of 12-month death by 24 % and for 12-month stroke by 101 %. The present study validates prior findings and refines their estimates among patients with diabetes mellitus. In addition, we demonstrated that the score was negatively correlated with the time to stroke and the time to death during follow-up. Furthermore, we demonstrated that CHA_2_DS_2_-VASc score yielded a moderately high negative predictive value (NPV) of 88 % for identifying patients at low risk of 12-month death and a high NPV of 99 % for identifying patients at low risk for 12-month stroke. Similar observations were made by Melgaard et al. [24] in a population of patients with heart failure and no AF.

CHA_2_DS_2_-VASc score comprises a cluster of common cardiovascular risk factors associated with thromboembolism. It is possible it may identify underlying conditions that may lead to AF, stroke, or death during follow-up. It is reported to predict incident AF in patients without preexisting AF [25–27]. More importantly, CHA_2_DS_2_-VASc score is recognized to be related to mortality and ischemic stroke in patients without AF and stroke [28, 29], heart failure [24], acute coronary syndromes [25], undergoing CABG [30]. Interestingly, Podolecki et al. analyzing patients with acute coronary syndromes failed to show any discrimination performance of CHA_2_DS_2_-VASc score in predicting incident MI during follow-up [16]. This finding is in agreement with our observations. Moreover, cerebral atherosclerosis is reported to be more common in patients with higher CHADS_2_ score. Kim et al. [31] demonstrated that patients with higher CHADS_2_ score had more carotid (both extra- and intracranial) stenosis. This finding may, in turn, result in an elevated risk of atherothrombotic stroke even in the absence of AF.

Overall, CHA_2_DS_2_-VASc score seems to be a well-known, simple tool that (1) comprises clusters of conditions that are associated with poor outcomes, (2) may increase the risk of incident AF in patients without preexisting AF leading to thromboembolic stroke, and (3) may be associated with a more extensive atherosclerosis leading to atherothrombotic stroke. All of the aforementioned mechanisms indicate the role of CHA_2_DS_2_-VASc score in predicting an increased morbidity and mortality. Taken together, these findings further support the potential role of CHA_2_DS_2_-VASc score in identifying high-risk patients that may require more aggressive management strategies.

## Strengths and limitations

We investigated a real-life population of diabetic patients with STEMI in which we did not exclude patients with severe comorbidities, including cardiogenic shock. These comorbidities may have predisposed some of the patients to a greater risk of stroke. Single-center design has also its shortcomings. We cannot exclude that some of the patients have had undiagnosed AF, as vascular disease (acute MI in this instance) is associated with a higher AF prevalence. Moreover, we lack data on the development of AF during follow-up, which may have resulted in the increased risk of stroke. In any way, we were able to demonstrate the role of CHA_2_DS_2_-VASc score in identifying patients at risk of increased 12-month mortality and stroke regardless the underlying mechanisms. Given the relatively low study size, however, these promising results should be verified on much larger cohorts.

## Conclusions

In diabetic patients with STEMI and no previous AF, median CHA_2_DS_2_-VASc score was high (4 points) and predicted 12-month death and stroke. However, it failed to predict in-hospital death and 12-month MI. CHA_2_DS_2_-VASc score had a similar discrimination performance in predicting 12-month mortality as TIMI risk score and a better discrimination performance in predicting 12-month stroke than TIMI risk score. Thus, it can serve as an additive tool in identifying high-risk patients that require aggressive management.
